# The contribution of endothelial cells to tissue fibrosis

**DOI:** 10.1097/BOR.0000000000000963

**Published:** 2023-09-07

**Authors:** Eloisa Romano, Irene Rosa, Bianca Saveria Fioretto, Mirko Manetti

**Affiliations:** Department of Experimental and Clinical Medicine, University of Florence, Florence, Italy

**Keywords:** endothelial cells, endothelial-to-myofibroblast transition, fibrosis, myofibroblasts, profibrotic mechanisms

## Abstract

**Purpose of review:**

Tissue fibrosis is an increasingly prevalent condition associated with various diseases and heavily impacting on global morbidity and mortality rates. Growing evidence indicates that common cellular and molecular mechanisms may drive fibrosis of diverse cause and affecting different organs. The scope of this review is to highlight recent findings in support for an important role of vascular endothelial cells in the pathogenesis of fibrosis, with a special focus on systemic sclerosis as a prototypic multisystem fibrotic disorder.

**Recent findings:**

Although transition of fibroblasts to chronically activated myofibroblasts is widely considered the central profibrotic switch, the endothelial cell involvement in development and progression of fibrosis has been increasingly recognized over the last few years. Endothelial cells can contribute to the fibrotic process either directly by acting as source of myofibroblasts through endothelial-to-myofibroblast transition (EndMT) and concomitant microvascular rarefaction, or indirectly by becoming senescent and/or secreting a variety of profibrotic and proinflammatory mediators with consequent fibroblast activation and recruitment of inflammatory/immune cells that further promote fibrosis.

**Summary:**

An in-depth understanding of the mechanisms underlying EndMT or the acquisition of a profibrotic secretory phenotype by endothelial cells will provide the rationale for novel endothelial cell reprogramming-based therapeutic approaches to prevent and/or treat fibrosis.

## INTRODUCTION

Connective tissue remodeling is a normal and crucial step of wound healing, but in pathological conditions, it can evolve in an irreversible fibrotic response characterized by a sustained activation of fibroblasts into myofibroblasts, with abnormal and excessive extracellular matrix (ECM) deposition and consequent increase in tissue stiffness, which further activates fibroblasts in a kind of vicious circle [1,2]. By profoundly affecting tissue structure and eventually culminating into organ failure, pathological fibrosis represents an important cause of global morbidity and mortality [1,2]. Fibrotic disorders, comprising a wide spectrum of diseases, including kidney, cardiac and lung fibrosis, inflammatory bowel disease-associated intestinal fibrosis, as well as fibroproliferative vascular remodeling in pulmonary arterial hypertension (PAH) and multisystem fibrosis in systemic sclerosis (SSc, or scleroderma), represent a major health problem due to the great increasing number of affected individuals and the void of effective disease-modifying therapeutic approaches [3–9].

Despite substantial heterogeneity in the cause and clinical manifestations of fibrotic diseases, several studies have recognized chronically activated fibroblasts (i.e., myofibroblasts) as the main cell population responsible for the accumulation of fibrotic scar tissue in multiple organs. Nevertheless, an increasing body of evidence highlights that vascular endothelial cells may significantly participate in the development and progression of fibrosis as well [1–4,10,11]. In particular, the contribution of endothelial cells to fibrosis has been demonstrated to be either direct, through an increase in the myofibroblast pool via endothelial transformation and concomitant microvascular rarefaction, or indirect, such as through the acquisition of a senescence-associated secretory phenotype (SASP) with paracrine release of profibrotic factors capable of activating tissue-resident fibroblasts, as well as proinflammatory mediators recruiting inflammatory/immune cells that, in turn, further promote the fibrotic process [1–4,10,11].

Among the variety of conditions featuring tissue fibrosis, SSc is a complex rheumatic disease that can be considered a prototypic multisystem fibrotic disorder, and a unique model to investigate the relationship of endothelial cells to fibrosis [4,12,13]. Indeed, the pathogenesis of SSc is characterized by early endothelial cell activation/damage and microvascular abnormalities that progressively evolve into myofibroblast-orchestrated untreatable fibrosis affecting the skin and multiple internal organs [4,11–13]. More specifically, in SSc, injured endothelial cells become dysfunctional, thus being unable to promote angiogenesis and vascular repair, promote tissue inflammation by enhancing the recruitment of circulating inflammatory/immune cells, contribute to the production of mediators responsible for fibrotic remodeling of the vascular wall and surrounding tissue, and directly transdifferentiate into profibrotic myofibroblasts [4,11,13,14].

Here, we will provide a brief overview of the different cellular and molecular mechanisms through which endothelial cells may participate in the development and progression of tissue fibrosis, with a particular focus on the most recent studies on fibrotic disorders such as SSc. 

**Box 1:**
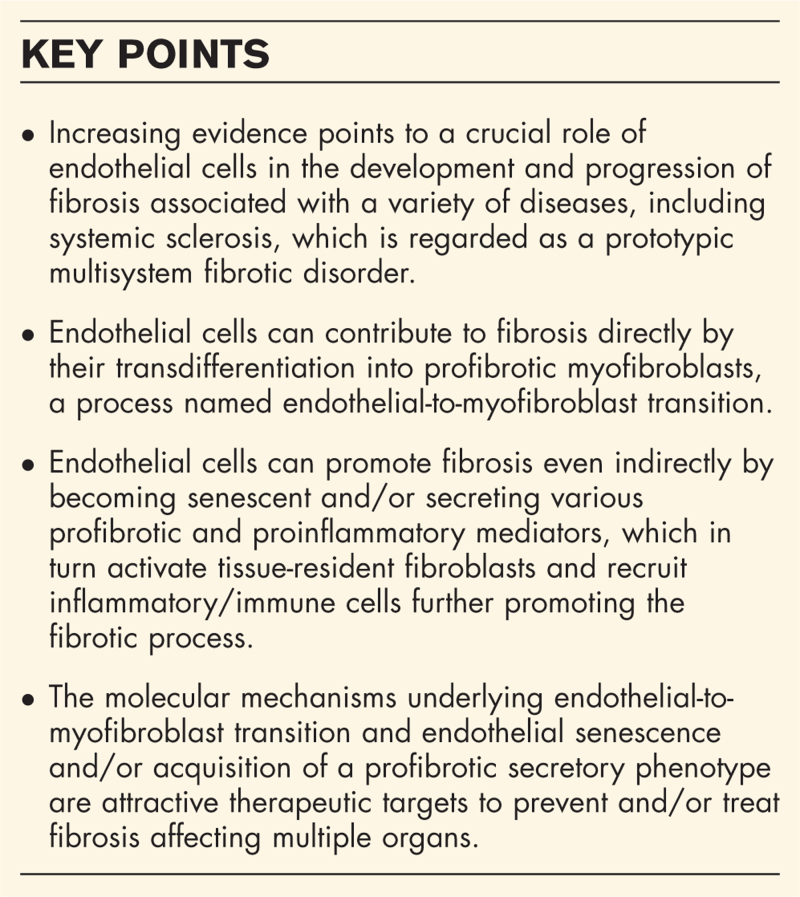
no caption available

## DIRECT CONTRIBUTION OF ENDOTHELIAL CELLS TO TISSUE FIBROSIS: THE ENDOTHELIAL-TO-MYOFIBROBLAST TRANSITION PROCESS

Endothelial cells constitute the inner lining of all blood vessels, thus representing a barrier between the bloodstream and tissues able to maintain vessel wall integrity [15,16]. In adult tissues, endothelial cells are in a quiescent state, while in case of insults such as tissue injury or ischemia, they can be induced to proliferate, migrate, and perform angiogenesis [1,15,16]. They also participate in coagulation, inflammatory/immune responses, and maintenance of blood pressure [1,15,16]. In addition to such a wide variety of cellular processes, more recently, endothelial cells have been demonstrated to be able to transdifferentiate into ECM-synthesizing myofibroblasts via a process named endothelial-to-myofibroblast transition (EndMT) [17–19]. During EndMT, endothelial cells detach from the endothelial layer, lose their polarity and cobblestone-like morphology reorganizing their cytoskeleton and turning into spindle-shaped fibroblast-like cells, and start to migrate into the surrounding tissue. Such a morphological change is accompanied by a phenotypic switch driven by the stabilization and nuclear translocation of the transcriptional regulator Snail1, and characterized by a downregulation of their specific markers CD31, von Willebrand Factor (vWF), and vascular endothelial-cadherin, paralleled by an upregulation of myofibroblast markers, including α-smooth muscle actin (α-SMA), S100A4/fibroblast-specific protein-1, type I collagen, and N-cadherin [17–19]. EndMT onset and progression may be promoted by several cytokines and growth factors, such as transforming growth factor (TGF)-β, interleukin (IL)-1β, tumor necrosis factor (TNF)-α, endothelin-1 (ET-1), as well as by different signaling pathways including those of Notch and Wnt [11,14,17–19]. Other putative mechanisms such as oxidative stress, disturbed shear stress, matrix stiffness, metabolic dysregulation, hypoxia, and epigenetic modifications also activate the EndMT process [11,14,15,17–20].

In recent years, EndMT has emerged as an important player in the development of tissue fibrosis associated with a variety of diseases, as well as an attractive target for therapeutic purposes [3,5–9]. For instance, in a very recent study employing a rat model of Crohn's disease-related intestinal fibrosis, the traditional Chinese herb Xue-Jie-San has been shown to be effective in repressing EndMT, as reflected by the upregulation of CD31 and vascular endothelial-cadherin and the parallel downregulation of S100A4 and α-SMA expression in the colon of 2,4,6-trinitrobenzene sulfonic acid-treated animals [5].

In two different mouse models of kidney injury/fibrosis, the conditional deletion of genes encoding Twist or Snail1 in endothelial cells inhibited the onset of EndMT and improved kidney fibrosis, limiting peritubular vascular leakage, reducing tissue hypoxia, and preserving tubular epithelial health and function [21]. In another study, endothelial expression of SIRT3 was found to be a crucial regulator of metabolic reprogramming and fibrogenesis in the kidneys of diabetic mice [22]. Indeed, endothelial cell-specific gain of function of the *SIRT3* gene by overexpression in a fibrotic mouse strain conferred resistance against diabetic kidney fibrosis, whereas selective SIRT3 loss of function in endothelial cells exacerbated renal collagen deposition [22]. Of note, in renal endothelial cells of SIRT3 transgenic mice, the expression of S100A4 and α-SMA was significantly reduced compared with diabetic control mice, whereas SIRT3 knockout mice showed higher endothelial levels of S100A4 and α-SMA, indicating that SIRT3 regulates EndMT-mediated activation of the fibrogenic pathways in the diabetic kidney [22]. A decreased expression of the endothelial marker CD31 and an increased expression of α-SMA were also reported in glomerular endothelial cells of patients and rats with diabetic kidney disease [23,24].

A significant role of EndMT in cardiac fibrosis has been demonstrated by the evidence that endothelial cells are able to differentiate into profibrotic myofibroblasts after myocardial infarction [25]. In a very recent study, lactate was found to promote cardiac fibrosis by regulating EndMT in the heart following myocardial infarction in mice [26]. In particular, inhibition of lactate production ameliorated myocardial infarction-induced EndMT, cardiac fibrosis, and cardiac dysfunction, while the administration of supplemental lactate further induced EndMT and worsened cardiac dysfunction [26]. *In vitro*, endothelial cells treated with lactate acquired mesenchymal-like functions, further suggesting that lactate may act as an important trigger of EndMT [26]. Furthermore, among the epigenetic mechanisms, the histone demethylase Jumonji domain-containing protein 2B has been identified as an important regulator of EndMT in a mouse model of myocardial infarction [27].

Concerning pulmonary fibrosis, which is the end-stage consequence of various forms of interstitial lung disease (ILD), changes in the structure of arterial layers with significant collagen and elastin deposition in the adventitia was found in patients with idiopathic pulmonary fibrosis (IPF) [28]. In addition, increased expression of the myofibroblast markers N-cadherin, S100A4, and vimentin was reported in the arterial layers of IPF patients, suggesting that resident lung endothelial cells may transdifferentiate into mesenchymal/myofibroblast-like cells [28]. In a recent study employing single-cell analysis and gene expression profile data from lung tissues of IPF patients, the number of endothelial cells was found to be significantly decreased, while the number of fibroblasts and myofibroblasts significantly increased [29]. Moreover, gene expression profile in the IPF group revealed an increase in the biological processes related to fibroblast function (e.g., cellular response to fibroblast growth factor stimulation and collagen fibril organization), suggesting the occurrence of EndMT during IPF development [29]. In another recent experimental work, endothelial-specific overexpression of sterol regulatory element-binding protein 2 (SREBP2), a protein with key role in oxidative stress-induced endothelial dysfunction, was shown to exacerbate vascular remodeling and induce pulmonary EndMT in bleomycin-treated mice [30]. Of note, SREBP2 was found to be highly expressed in the lungs of IPF patients, suggesting that this protein can aggravate pulmonary fibrosis by promoting EndMT in pulmonary microvessels [30]. Interestingly, it has also been recently hypothesized that SARS-CoV-2-induced endothelial dysfunction might induce post-COVID pulmonary fibrosis and vascular remodeling through the EndMT process [31,32].

PAH is a complex and progressive disease characterized by the abnormal remodeling of the pulmonary arteries resulting in right ventricular failure and death [9,33]. In in-vitro studies, TGF-β treatment of pulmonary arterial endothelial cells has been shown to induce the loss of endothelial markers and the concomitant acquisition of mesenchymal markers such as α-SMA and vimentin, suggesting an ongoing EndMT process [34]. Hypoxia was also found to induce pulmonary EndMT, as an upregulation of hypoxia-inducible transcription factor (HIF)-1α and HIF-2α was demonstrated in lung tissues and isolated pulmonary arterial endothelial cells from patients with idiopathic PAH and three different rodent models of PAH [35]. HIF-1α knockdown was also found to block hypoxia-induced EndMT in pulmonary microvascular endothelial cells [36]. Apart from transcription factors, also microRNAs were demonstrated to be implicated in EndMT in PAH. Indeed, the overexpression of miR-181b in pulmonary arterial endothelial cells explanted from a rat model of PAH was able to dampen inflammation-induced EndMT by downregulating the expression of TGF-β receptor 1 (TGF-βR1) and circulating levels of the proteoglycan endocan [37]. Finally, the loss of bone morphogenetic protein receptor type 1A in endothelial cells was found to induce elevated expression of TGF-βR2, thus promoting EndMT, pulmonary vascular remodeling, and PAH in mice [38].

A growing body of evidence supports a pivotal role of EndMT in SSc as well, particularly in its main clinical manifestations, including dermal fibrosis, ILD, and PAH [14,39–44]. In the skin, endothelial cells in intermediate stages of EndMT have been found in dermal microvessels of both SSc patients and two different experimental mouse models of SSc, namely, the bleomycin-induced skin fibrosis and the urokinase-type plasminogen activator receptor (uPAR)-deficient mouse models [40]. Similarly, dermal microvascular endothelial cells explanted from the involved skin of SSc patients were reported to express both endothelial and myofibroblast markers and to display a spindle-shaped morphology and a contractile phenotype in culture [40]. Of note, also, healthy dermal microvascular endothelial cells challenged with SSc sera acquired a myofibroblast-like appearance and the ability to contract *in vitro*, an effect that was demonstrated to be partly induced by the cleavage of uPAR by serum matrix metalloproteinase (MMP)-12 [40]. Interestingly, MMP-12 is known to be significantly augmented in SSc serum and tissues [45], and such an upregulation has been suggested to be induced by SSc fibroblast-mediated extracellular acidosis [46]. Indeed, highly glycolytic SSc skin fibroblasts have been reported to create an acidic milieu able to trigger MMP-12 overexpression and the subsequent truncation of uPAR on endothelial cells, thus promoting EndMT [46]. In another in-vitro study, microvascular endothelial cells from SSc-unaffected skin co-cultured with fibroblasts from SSc-affected skin underwent EndMT in the presence of ET-1 and TGF-β [47]. Interestingly, such an ET-1/TGF-β-mediated EndMT process has been confirmed in the skin and lungs of a mouse model of tissue fibrosis induced by TGF-β [48]. Furthermore, in the experimental SSc model of KLF5+/−;Fli1+/− mice, isolated dermal endothelial cells displayed a reduced expression of the endothelial markers vascular endothelial-cadherin and CD31, suggesting that KLF5 transcription factor deficiency may participate in the induction of EndMT in SSc [49]. Other molecules which have been proposed to be possible drivers of SSc-related EndMT are caveolin-1, fibrillin-1, and interferon regulatory factor-5 [11]. More recently, the occurrence of EndMT in SSc has been related to elevated levels of oncostatin M, a member of the IL-6 family, and the inflammatory lipid mediator leukotriene B4, with the former being able to induce myofibroblast-like morphologic changes in healthy dermal microvascular endothelial cells, and the latter to promote the myofibroblast transition in endothelial cells through the activation of the phosphatidylinositol 3-kinase/protein kinase B (AKT)/mammalian target of the rapamycin (mTOR) pathway [50,51]. Interestingly, the inhibition of AKT/mTOR signaling in different ways, namely by the iridoid glycoside geniposide, the phytochemical drug tanshinone IIA, and the bone morphogenic protein-7, was shown to significantly dampen EndMT, thus exerting antifibrotic effects, in both cultured endothelial cells and bleomycin-induced scleroderma mouse model [52,53,54^▪^]. In a very recent study, using unbiased transcriptome analysis of SSc skin biopsies, the downregulation of SPAG17 has been recognized as an additional driver of sustained EndMT [55^▪^]. Of note, the same authors also demonstrated that mice lacking SPAG17 displayed spontaneous skin fibrosis, and that SPAG17 knockdown in microvascular endothelial cells was accompanied by spontaneous myofibroblast differentiation [55^▪^]. In another study, the overexpression of the chemokine CXCL4, which is known to be increased and strongly correlated with skin and lung fibrosis in SSc, was reported to induce EndMT in in-vitro cultures of endothelial cells and to aggravate skin, lung, and cardiac fibrosis in mice [56]. Finally, different studies have focused on the possible effects of some therapeutic compounds on SSc-related EndMT [57–59]. In particular, both systemic and topical administration of dihydroartemisinin, a drug employed in the treatment of malaria, was able to reduce dermal thickness and collagen deposition, as well as to diminish EndMT in mouse models of skin fibrosis. This molecule was also able to partially counteract the profibrotic effect of TGF-β1 on endothelial cells *in vitro*[57]. Iloprost, a synthetic analogue of prostacyclin broadly used for the treatment of SSc, was reported to significantly inhibit EndMT in human healthy dermal endothelial cells challenged with TGF-β and in SSc dermal endothelial cells [58]. Moreover, treatment of microvascular endothelial cells explanted from SSc skin with a synthetic stimulator of the soluble guanylate cyclase resulted in EndMT suppression by blunting the myofibroblast-like profibrotic phenotype of SSc dermal endothelial cells [59].

Apart from in the skin, the presence of endothelial cells in intermediate stages of EndMT has been identified also in the lungs of SSc patients, with myofibroblast transdifferentiation of ECs contributing to both SSc-related PAH and ILD [11,14,41,60,61]. Indeed, the co-expression of endothelial and myofibroblast markers has been demonstrated in the pulmonary arterioles of SSc patients with PAH, in an experimental mouse model of hypoxia-induced PAH, and in lung tissue of SSc patients with ILD [61–63]. A recent microarray analysis confirmed the presence of EndMT in SSc-ILD by reporting elevated expression of myofibroblast-specific genes in lung microvascular endothelial cells [64]. In a genetic lineage tracing mouse model used to investigate the fate of endothelial cells, it has been observed that, after treatment with bleomycin, lung endothelial cells undergo only a partial EndMT characterized by an increased expression of myofibroblast markers but no changes in endothelial markers [65]. Furthermore, the same authors demonstrated that macrophage depletion, in combination with bleomycin injection, was able to upregulate genes associated with EndMT in pulmonary endothelial cells, highlighting the possible contribution of lung macrophages in preventing EndMT-mediated fibrosis [65]. In another study performed on human healthy pulmonary microvascular endothelial cells, stimulation with SSc sera increased reactive oxygen species (ROS) production and promoted EndMT by leading to collagen and α-SMA overexpression and vWF downregulation [66]. As these effects were prevented by the cell exposition to the NADPH oxidase inhibitor diphenyleneiodonium, it has been hypothesized a causative role of ROS in EndMT [66]. Finally, a very recent study evaluating the protective effects of linagliptin, a highly specific dipeptidyl peptidase-4 inhibitor, on bleomycin-induced pulmonary fibrosis, revealed that this molecule was able to attenuate EndMT both *in vivo* and *in vitro*[67].

Last but not least, it is noteworthy to underline that in SSc, the EndMT process may play different pathogenetic roles and, hence, be associated with diverse clinical manifestations depending on the type of vessels involved [14]. Indeed, if in arterioles and small arteries, it may determine myofibroblast accumulation within the vessel wall, with the consequent onset of a “fibroproliferative vasculopathy” (i.e., thickening of the vessel wall with occlusive vascular disease) as observed in SSc-related PAH, when occurring in thin-walled capillaries, it may instead lead to an increased amount of perivascular ECM-synthetizing myofibroblasts and a concomitant loss of endothelial cells, thus representing an important link between tissue fibrosis and “destructive vasculopathy” characterized by microvessel rarefaction as largely documented in SSc skin [14].

## INDIRECT CONTRIBUTIONS OF ENDOTHELIAL CELLS TO TISSUE FIBROSIS

Apart from directly transdifferentiating into myofibroblasts, endothelial cells may contribute to tissue fibrosis also in different indirect ways comprising the acquisition of a senescent phenotype and the release of exosomes and a variety of profibrotic/proinflammatory mediators, with consequent recruitment of inflammatory/immune cells that further boost the fibrotic process [1,68–70].

Senescent cells are cell cycle arrested cells characterized by morphological and metabolic changes, chromatin reorganization, and altered gene expression [68,69]. Indeed, they display heterogeneous markers of senescence such as the proliferation inhibitors p16INK4A, p21, or p53, and a proinflammatory secretory phenotype, known as SASP, encompassing a variety of biologically active mediators, including growth factors and cytokines (e.g., TGF-β, TNF-α, IL-1β, and IL-6), chemokines, ECM proteins, and enzymes (e.g., MMPs) [68,69]. Through these secreted factors, senescent cells signal in both autocrine and paracrine ways, likely inducing senescence in neighboring cells and allowing senescence to spread [68,69]. Programmed cellular senescence seems to be an apoptosis-like transient biological mechanism that can be exploited to eliminate unwanted cells, as the inflammatory SASP leads to the recruitment of immune cells with consequent cell clearance. For example, during wound healing, such a process contributes to the elimination of myofibroblasts and the resolution of the tissue remodeling process. However, some senescent cells appear to be able to escape clearance and accumulate within tissues, further dysregulating neighboring cells through their SASP and thus contributing to promote fibrosis [68,69]. As far as endothelial cells are concerned, when undergoing cellular senescence, they display characteristic alterations in gene expression associated with functional changes, including lower proliferative rates, decreased activity of endothelial nitric oxide synthase, and defective angiogenic capacities [68,69]. Moreover, senescent endothelial cells have also been suggested to preferentially undergo EndMT, thus contributing even directly to organ fibrosis [71^▪▪^].

There is accumulating evidence that endothelial senescence is elevated in fibrotic diseases. In a very recent study, single-cell RNA sequencing was used to create a transcriptome atlas of murine renal endothelial cells of young and aged mice and, thus, identify transcriptomic changes occurring during aging [72]. Among the different subtypes of renal endothelial cells, Pi16+ glomerular and Sparcl1+ angiogenic endothelial cells were found to be the most affected by senescence, and a chronic state of inflammation and compromised glomerular function was revealed as a prominent aging feature [72]. In addition, a high proinflammatory microenvironment was observed in aged glomerular endothelial cells, allowing the authors to suppose that such a milieu may contribute to age-related renal fibrosis [72]. In a mouse model of chronic cardiac pressure overload, endothelial cell-specific senescence inhibition through p53 deletion was shown to reduce vascular rarefaction and fibrosis [73], while cardiovascular disease risk factors such as aging, obesity, hypertension, and physical inactivity were found to induce senescence and a mesenchymal/myofibroblast-like phenotype in mouse cardiac endothelial cells [74]. Interestingly, the deacetylase SIRT1 has been found to suppress endothelial cell senescence by deacetylating and inactivating p53 [1], and its endothelial cell-specific overexpression has been proved to be able to inhibit TGF-β-induced EndMT and to attenuate isoproterenol-induced cardiac fibrosis in mice [75].

A potential role of endothelial cell senescence has been explored also in pulmonary fibrosis. For instance, by using a unique endothelial cell-specific progeroid mice, some authors recently revealed a detrimental role of senescent endothelial cells in the progression of IPF, potentially via the acceleration of EndMT and the enhancement of the myofibroblastic transition of resident lung fibroblasts through their SASP [71^▪▪^]. In addition, by performing an epigenetic and transcriptional analysis of lung endothelial cells from young and aged mice during the resolution or progression of bleomycin-induced lung fibrosis, other researchers demonstrated that the transcription factor ETS-related gene (ERG) is a putative orchestrator of lung capillary homeostasis and repair [76]. In fact, ERG function was found to be dysregulated in aging, and its loss both enhanced paracrine fibroblast activation *in vitro* and impaired lung fibrosis resolution in young mice *in vivo*[76]. As far as SSc is concerned, an association with senescence was shown at a genomic level, as whole exome sequencing of microdissected areas of dermal fibrosis in skin biopsies from patients with severe skin and lung involvement revealed the presence of several somatic mutations with a clock-like senescence signature [77^▪▪^]. Of note, sera from SSc patients containing disease-specific autoantibodies (i.e., anti-CENP-B and anti-TOPO-1 autoantibodies) have been shown to induce vascular endothelial cell senescence *in vitro*[78]. Moreover, another in-vitro study provided the first demonstration of the pathogenicity of immunocomplexes with different SSc-specific autoantibodies on endothelial cells [79]. In fact, SSc immunocomplexes triggered endothelial cell activation that, in turn, led to the acquisition of a profibrotic phenotype in healthy skin fibroblasts [79].

Increasing literature also suggests that endothelial cell-derived exosomes can contain profibrotic factors able to exert strong molecular and gene expression effects on different target cells, resulting in their phenotypic conversion into activated myofibroblasts [70]. In SSc, the effects of exosomes isolated from SSc sera on gene expression patterns of normal dermal fibroblasts were examined, revealing that they were able to induce a profibrotic phenotype in such cells [80]. Although such an effect was not investigated on other cell types, it is likely that a similar mechanism may also activate endothelial cells, eventually resulting in their transdifferentiating into myofibroblasts.

## CONCLUSION

**FIGURE 1:**
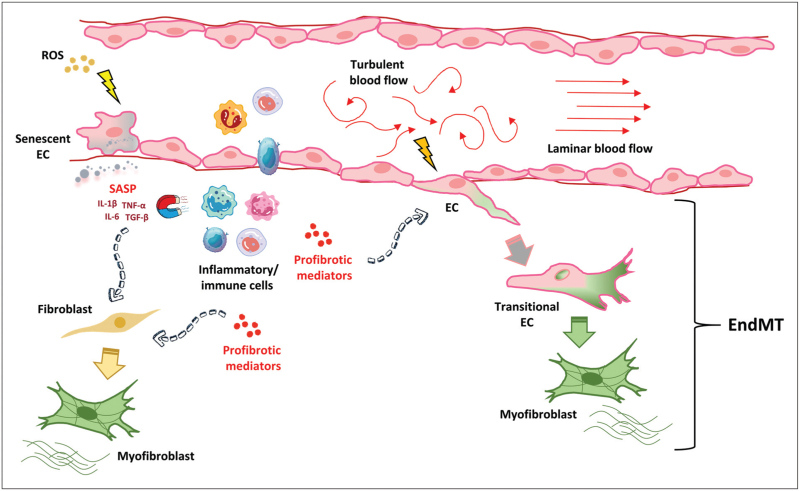
Schematic overview of endothelial cell involvement in tissue fibrosis. ECs mainly contribute to the fibrotic process by acting as a source of myofibroblasts through the EndMT process, which is supported by inflammation, low shear stress or disturbed flow stress, as well as EC senescence. ECs may also participate to fibrosis in multiple indirect ways by becoming senescent and/or secreting a variety of profibrotic and proinflammatory mediators with consequent fibroblast activation and recruitment of inflammatory/immune cells that further trigger fibrosis. EC, endothelial cell; EndMT, endothelial-to-myofibroblast transition; IL, interleukin; ROS, reactive oxygen species; SASP, senescence-associated secretory phenotype; TGF-β, transforming growth factor-β; TNF-α, tumor necrosis factor-α.

Increasing evidence points out that endothelial cells play a pivotal role in the development and progression of the fibrotic process associated with multiple diseases including SSc. In particular, endothelial cells mainly contribute to fibrosis directly differentiating into profibrotic myofibroblasts through a process named EndMT (Fig. 1). Endothelial cells can also promote fibrosis indirectly by becoming senescent and/or secreting various profibrotic and proinflammatory mediators, which in turn activate tissue-resident fibroblasts and recruit inflammatory/immune cells further promoting the fibrotic process (Fig. 1). Future research aimed at unveiling the molecular mechanisms that underly EndMT and the acquisition of a senescent phenotype by endothelial cells has the great potential to pave the way for the development of new therapeutic strategies able to prevent and/or treat fibrosis of multiple organs.

## Acknowledgements

*None.*

### Financial support and sponsorship

*None.*

### Conflicts of interest

*There are no conflicts of interest.*

## REFERENCES AND RECOMMENDED READING

Papers of particular interest, published within the annual period of review, have been highlighted as:

▪ of special interest
▪▪ of outstanding interest
